# Phytoremediation Potential of Maná-Cubiu (*Solanum sessiliflorum* Dunal) for the Deleterious Effects of Methylmercury on the Reproductive System of Rats

**DOI:** 10.1155/2014/309631

**Published:** 2014-03-19

**Authors:** Raquel Frenedoso da Silva, Gabriela Missassi, Cibele dos Santos Borges, Eloísa Silva de Paula, Maria Fernanda Hornos Carneiro, Denise Grotto, Fernando Barbosa Junior, Wilma De Grava Kempinas

**Affiliations:** ^1^Department of Morphology, Institute of Biosciences, Univ Estadual Paulista-UNESP, Distrito de Rubião Junior s/n**º**, 18618-970 Botucatu, SP, Brazil; ^2^Department of Clinical Analyses, Toxicology and Food Sciences, School of Pharmaceutical Sciences of Ribeirão Preto, University of São Paulo, Avenida do Café s/n**º**, 14040-903 Ribeirão Preto, SP, Brazil

## Abstract

Methylmercury, organic form of mercury, can increase the number of abnormal sperm and decrease sperm concentration and testosterone levels possibly due to the damage caused by reactive species to germ and Leydig cells. Maná-cubiu (*Solanum sessiliflorum* Dunal) is a native fruit from Amazon rich in iron, zinc, niacin, pectin, and citric acid, used in foods, beverages, and medicinal purposes, since it has been useful for treatment of various diseases caused by oxidative stress or nutritional deficiency. Therefore, this study evaluated the phytoremediation potential of this fruit on damages caused by exposure to MeHg on sperm quantity and quality and the histological aspect of the testis and epididymis. Wistar male rats (*n* = 20) were randomly allocated into four groups: Control group (received distilled water), MeHg group (140 *μ*g/Kg), *Solanum* group (1% of fruit Maná-cubiu on chow), and *Solanum* plus MeHg group (same treatment as MeHg and Solanum group). The organs were weighted, histopathology; sperm morphology and counts were obtained. The results showed reduction in body weight gain, testis weights, reduced sperm production, and increased histopathological abnormalities in the MeHg-treated group. However, treatment with *Solanum* plus MeHg revealed a protective effect of this fruit on damages caused by MeHg.

## 1. Introduction

Mercury (Hg) is a trace metal widely used and marketed for centuries and may be found in air, water, foods of plant and animal origin, and even in pharmaceuticals such as vaccines [1, 2]. Sulfate-reducing bacteria present in rivers, lakes, and oceans methylate inorganic Hg, transforming it into its organic form methylmercury (MeHg) [3], which can be transferred to humans through the food chain, mainly by eating fish predators.

Cellular damage caused by MeHg is mainly due to increased production of reactive oxygen species (ROS) that causes oxidative stress [4]. In addition, interactions occur between Hg and thiol groups of proteins, forming complexes that bind to important proteins as glutathione, cysteine, and superoxide dismutase, inactivating them, reducing the antioxidant defenses of cells [5, 6]. Glutathione is a major suppressor of ROS, being the result of a complex mechanism that defends the cell from oxidative damage. Its reduced form, the reduced gluthatione (GSH), is able to eliminate the reactive species molecules and plays central role in maintenance of redox state of the cell [7, 8].

Hg has proven deleterious effects on male reproductive system that have been extensively investigated over the past years [9, 10]. The oral exposure of rats to mercury derivatives reduces the serum testosterone levels [2], testicular weight, and sperm production, besides causing DNA fragmentation [9]. A study of our research group showed that changes caused by exposure to MeHg, such as decreased sperm concentration and increase of sperm with abnormal head, can be attributed to low levels of testosterone [11]. The reduction in the levels of this hormone may be due to damage caused by reactive species to Leydig cells, impairing its synthesis in the testis of exposed animals [12].

It has been demonstrated that consumption of antioxidants present in certain foods can prevent toxic effects caused by exposure to trace metals, once it protects the cell from DNA damage and changes its redox state induced by MeHg [5]. In this sense, the maná-cubiu (*Solanum sessiliflorum *Dunal), a native fruit from the Amazon, is often used in foods and beverages as well as in the production of cosmetics and plant-derived medicines due to the antioxidant properties of its bioactive compounds—iron (Fe), selenium (Se), manganese (Mn), zinc (Zn), citric acid, carotenoids, and phenolic compounds [13, 14]. Marx et al. [15] present a detailed analysis of the main components of the maná-cibiu fruit.

Despite the antioxidant health effects attributed to its consumption, there are no studies regarding the effects of the maná-cubiu on the MeHg toxicity in male reproductive system. Therefore, the objective of this work was to evaluate the phytoremediation potential of maná-cubiu on the damages caused by chronic exposure to MeHg in the rat sperm parameters and the histological aspect of the testis and epididymis.

## 2. Material and Methods

### 2.1. Animals

Wistar male rats weighting 100 g were supplied by the Central Laboratory Animal Breeding Center (University of São Paulo, Ribeirão Preto, Brazil) and allocated individually in polypropylene cages, with laboratory grade pine shavings as bedding. Rats were maintained under controlled temperature (22–25°C) and lighting conditions (12 L/12 D photoperiod). Rat chow and filtered tap water were provided* ad libitum*. Experimental procedures were in accordance with the guidelines of the Committee on Care and Use of Experimental Animal Resources, University of São Paulo, Brazil (Approved protocol number: 12.1.1599.53.8).

### 2.2. Experimental Design

The animals were randomly allocated into four groups (*n* = 5 per group): Control group, MeHg group,* Solanum* group, and* Solanum* plus MeHg group. The MeHg group rats received daily oral gavage doses of 140 *μ*g/Kg of CH_3_HgCl; the* Solanum* group rats received chow containing 1% of fruit pulp maná-cubiu lyophilized; the MeHg +* Solanum* group rats received the same treatment than group MeHg and* Solanum*; and the Control group rats received distilled water. The animals were treated for 100 days, weighted each 14 days, and, at the end of treatment, were euthanized with super doses of ketamine and xilasine. The following parameters were GSH level in total blood, body and reproductive organ weights, sperm morphology, sperm counts in the testis, and epididymis and testicular and epididymal histopathology.

### 2.3. Minerals Determination in* Solanum sessiliflorum* by ICP-MS

Before digestion of the fruit, 15 g was separated by quartering as described by [16] and divided into two plastic tubes. The contents of each tube were ground for 3 minutes in a ball mill (TECNAL TE 350, Brazil) and sifted in a 106 *μ*m sieve (BERTEL, Brazil). Then, samples were digested in closed vessels using a microwave oven decomposition system (MILESTONE ETHOS D, Italy) according to the method proposed by [17]. Briefly, fruit samples (250 mg) were accurately weighed in a PFA digestion vessel and then 5 mL of nitric acid 20% v/v + 2 mL of 30% (v/v) H_2_O_2_ were added. The vessel was placed inside the microwave oven and decomposition was carried out according to the following program: (a) 160°C (power of 1000 W) for 4.5 minutes; (b) 160°C (power of 0 W) for 0.5 minutes; (c) 230°C (power of 1000 W) for 20 minutes; and (d) 0°C (power of 0 W) for 20 minutes. After that, the solutions were left to cool and the volume was made up to 50 mL with Milli-Q water. Rhodium was then added as an internal standard to a final concentration of 10 *μ*g·L^−1^.

All measurements were conducted using an ICP-MS (Elan DRC II PerkinElmer, Norwalk, CT) with high-purity argon (99.999%, White Martins, Brazil), which used a Meinhard concentric nebulizer (Spectron/Glass Expansion, Ventura, CA) connected to a cyclonic spray chamber. A radiofrequency (rf) of 1200 W power was selected in pulse mode with autolens one. Sample data were acquired by using 20 sweeps/reading, 1 reading/replicate, and a dwell time of 50 ms. Argon nebulizer gas flow rate was optimized daily from 0.5 to 0.9 L/min. Data were acquired in counts per second (cps). The following isotopes were selected: Mn, Se, Fe, Zn, Co, and Mg.

### 2.4. Reduced Glutathione (GSH) Assay

Reduced thiols in total blood, represented by GSH quantification, were determined by the method of Ellman [18]. Blood (0.15 mL) was hemolyzed by 10% TritonX-100 (0.1 mL) and precipitated with 0.1 mL of TCA. After centrifugation at 3000 g and 4°C for 10 min, the supernatant aliquots were reacted to 50 mL of DTNB. The final reaction product was read at 412 nm in a spectrophotometer. GSH levels were expressed as millimoles per milliliter of blood.

### 2.5. Reproductive Organ Weights

Immediately after the euthanasia, testis, epididymis, ventral prostate, and seminal vesicle (without the coagulating gland) were removed and their wet weights (absolute and relative to body weight) were measured.

### 2.6. Sperm Morphology

Sperm were removed from the left vas deferens by internal rising with 1 mL formol-saline solution with the aid of a syringe and needle. To analyze sperm morphology, smears were prepared on histological slides that were left to dry for 90 minutes and observed in a phase-contrast microscope (400x magnification) [19]. Two hundred spermatozoa were analyzed per animal. Morphological abnormalities were classified into two general categories: head morphology and tail morphology [20]. Sperm were also classified as to the presence or absence of the cytoplasmic droplet.

### 2.7. Daily Sperm Production per Testis, Sperm Number, and Transit Time in the Epididymis

The right testis and epididymis were used for sperm counts. Homogenization-resistant testicular spermatids (stage 19 of spermiogenesis) in the testis were counted as described previously [21]. Briefly, the testis was decapsulated, weighed soon after collection, and homogenized in 5 mL NaCl 0.9% containing Triton X100 at 0.5%, followed by sonication for 30 s. After a 10-fold dilution, one sample was transferred to Neubauer chambers (four fields per animal), and late spermatids were counted. To calculate the daily sperm production (DSP), the number of homogenization resistant spermatids was divided by 6.1, the number of days these spermatids are present in the seminiferous epithelium. In the same manner, caput/corpus and cauda epididymidis portions were cut into small fragments with scissors and homogenized, and sperm were counted as described for the testis. The sperm transit time through the epididymis was determined by dividing the number of sperm in each portion by the DSP.

### 2.8. Histological Analysis of Testis and Epididymis

The left testis and epididymis were fixed in Bouin solution for 24 h. The pieces were dehydrated in a graded ethanol series and routinely processed for embedding in paraffin, sectioned at 5 *μ*m, and subsequently stained with hematoxylin and eosin (H&E). Testis and epididymis sections were examined by light microscopy following specific guidelines for toxicological studies [22].

### 2.9. Statistical Analysis

Data are presented as mean ± standard error of mean (SEM) for parametric variables (ANOVA test followed by Dunnett was used). For comparison of nonparametric variables Mann-Whitney test or Kruskal-Wallis followed by Dunn test was used and expressed as median and interquartile range. Differences were considered significant when *P* < 0.05. The statistical analyses were performed by GraphPadInStat (version 5).

## 3. Results

### 3.1. Minerals Determination in* Solanum sessiliflorum* by ICP-MS

The analysis performed showed that maná-cubiu fruit has the following chemical composition: Mn: 9.189 *μ*g/g; Se: 0.162 *μ*g/g; Fe: 36.952 *μ*g/g; Zn: 17.358 *μ*g/g. Also, the fruit presents cobalt (Co—0.054 *μ*g/g) and magnesium (Mg—2.815.518 *μ*g/g).

### 3.2. Reduced Glutathione (GSH) Assay

GSH quantification showed that MeHg reduced levels of this enzyme in blood.

**Figure 1:**
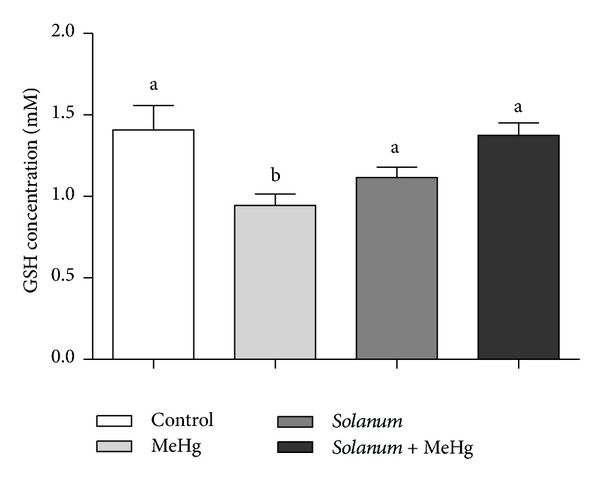
GSH quantification of control and treated animals. One-way analysis of variance (ANOVA) test followed by Tukey test was performed. ^a, b^Mean values with the same letter do not differ statistically; *P* values < 0.05 were considered significant.

On the other hand,* Solanum* + MeHg group showed GSH levels similar to Control group (Figure 1).

### 3.3. Body Weight Gain and Reproductive Organ Weights

**Figure 2:**
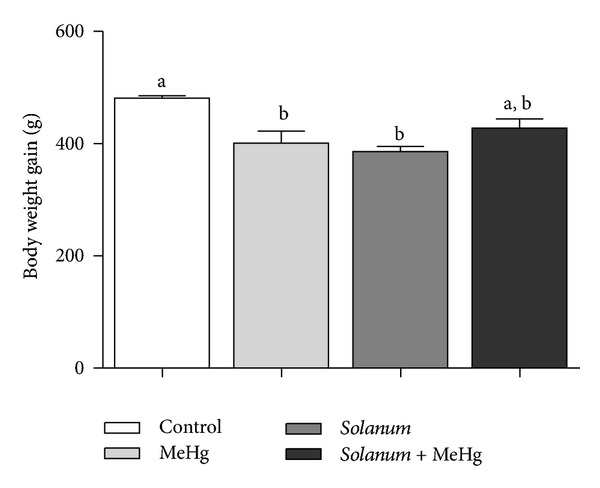
Weight gain of control and treated animals. Values expressed as mean ± SEM. One-way analysis of variance (ANOVA) test followed by Tukey test was performed. ^a, b^Mean values with the same letter do not differ statistically; *P* values <0.05 were considered significant.

Significant reductions in the body weight gain (Figure 2) and final body weight (Table 1) were reported in both MeHg and* Solanum*-treated groups. Also, the animals treated with MeHg showed a reduction in the testis weight. However, animals that received both treatments, that is,* Solanum* plus MeHg, showed comparable final body weight and testicular weight compared to the control group (Table 1).

### 3.4. Sperm Morphology

Analysis of sperm morphology revealed that MeHg group had lower percentage of morphologically normal spermatozoa, whereas the predominant abnormality was in the sperm head. Interestingly the group treated with MeHg plus* Solanum* showed no change in sperm morphology. A large amount of spermatozoa presented cytoplasmic droplet in all experimental groups, especially on* Solanum* + MeHg.* Solanum* group showed an improvement on flagellum morphology compared to other groups (Table 2).

### 3.5. Daily Sperm Production per Testis, Sperm Number, and Transit Time in the Epididymis

The treatment with MeHg decreased the DSP and epididymal sperm number compared to Control group. This harmful effect was prevented by the use of* Solanum*, as shown by the results of the group MeHg plus* Solanum* (Table 3).

### 3.6. Histological Analysis of Testis and Epididymis

**Figure 3:**
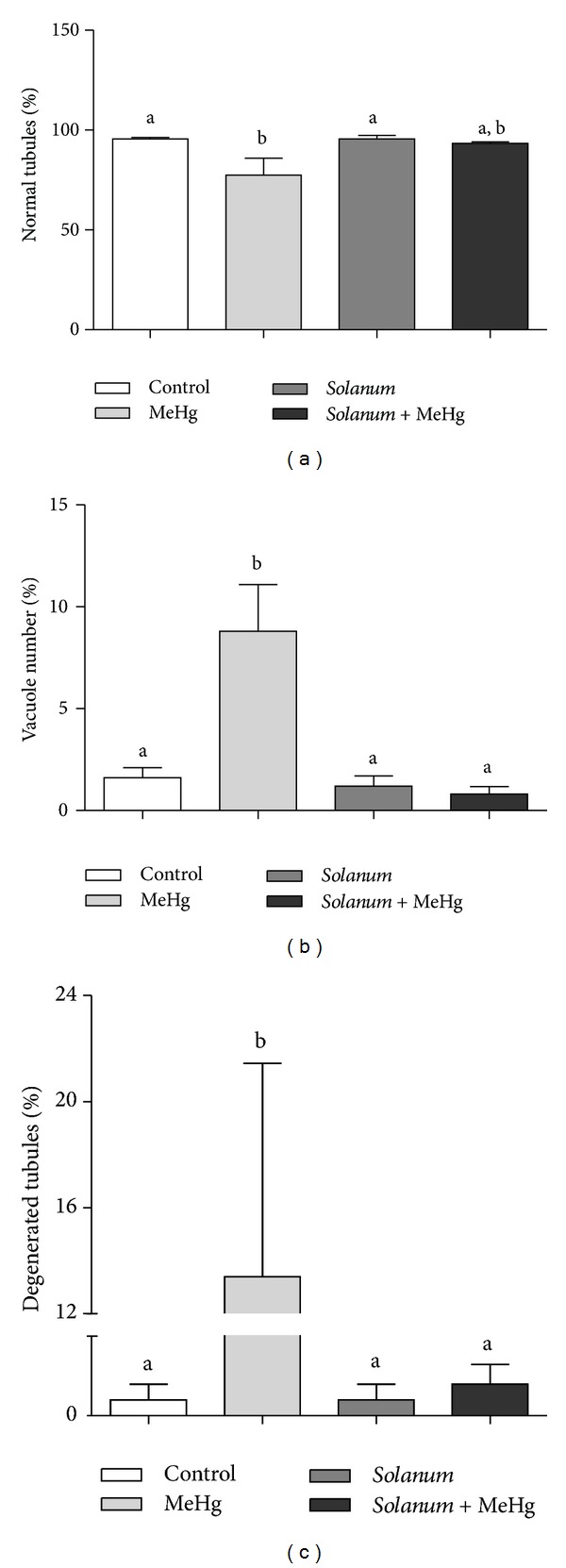
Histopathological evaluation of the testis. Values expressed as mean ± SEM. Kruskal-Wallis analysis of variance test followed by Dunn's test was performed. ^a, b^Mean values with the same letter do not differ statistically; *P*-values <0.05 were considered significant.

**Figure 4:**
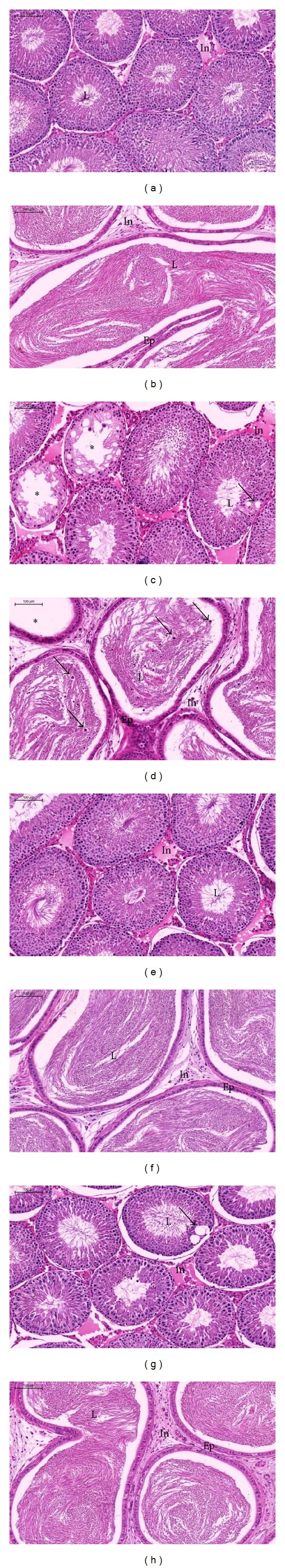
Histological aspect of the testis and cauda epididymis (*n* = 5/group): (a, b) control group, (c, d) MeHg group, (e, f)* Solanum* group, and (g, h) MeHg +* Solanum* group. GC: germ cells; L: lumen; In: interstitial tissue; Ep: epithelium. Observe the presence of vacuoles (arrow) and degenerated seminiferous epithelium (asterisk) in the testis and empty segments (asterisk) and cell bodies in the lumen (arrow) in the epididymis. Final magnification: 200x.

Histopathological evaluation showed a significant increase in the percentage of abnormal seminiferous tubules in the MeHg treated group relative to the Control and* Solanum* group. The abnormalities found were predominantly seminiferous tubules with vacuolization and degeneration (Figures 3 and 4). Epididymal evaluation showed an increased incidence of cell bodies in the lumen of the caput and cauda epididymis in the MeHg treated group (Figure 4).

## 4. Discussion

Methylmercury is the most toxic form of mercury due to its lipophilic properties, which enable it to overcome some cellular barriers [1]. Several studies have described its neurotoxic [23, 24], hepatotoxic [25, 26], and cardiotoxic action [27]. Also, this substance has deleterious effects on the male and female reproductive systems [9, 28].

Maná-cubiu presents several compounds in its composition that have relevant antioxidant properties as phenolic compounds, hydrophilic extracts, and carotenoids, which are capable of scavenging reactive species of oxygen and nitrogen [13], as well as chemical elements essential to assist in the proper diet as zinc and selenium [5, 14, 29]. Considering the constituents of maná-cubiu and their beneficial effects, the present study evaluated the phytoremediation potential of this fruit on the deleterious effects on rat sperm quantity and quality promoted by MeHg.

Changes in body weight are indicative signs of systemic toxicity [30] and its reduction may be related to the effects of exposure to toxic substances. There was a reduction in body weight gain of animals treated with MeHg, as shown previously by Fossato da Silva et al. [11]. Moreover, animals exposed to MeHg had decreased testis weights, possibly due to the toxic effects of MeHg on this organ. The determination of organ weights is an important parameter for assessing the risk of toxicity on the male reproductive system [30]. Although there was a reduction in the weight gain in the* Solanum*-treated group, the reproductive organ weights were comparable among groups.

The toxicity caused by MeHg is closely related to oxidative stress that develops as a consequence of an imbalance between excessive production of reactive species of oxygen (ROS) and impaired antioxidant defense system after exposure [31, 32]. These reactive species are bioproducts of oxygen metabolism, constantly produced in normal cells by mitochondria, especially by sperm during capacitation process, but its action is neutralized by cell antioxidant system [33]. However, when testicular and epididymal cells are exposed to harmful factors such as excessive heat, radiation, chemicals, or trace metals, there is an overproduction of these molecules potentially causing male infertility [34, 35], since ROS can cause damages to sperm function and DNA integrity [36], especially if the defense system is lagged. In this work, we showed that MeHg is capable of decreasing the GSH concentration present in blood of treated animals, as also observed by Barcelos et al. [5] and Grotto et al. [6], indicating that the toxicity is related to oxidative stress and to the imbalance of cellular antioxidant system. The protective antioxidant effect of* Solanum* is proved since GSH levels are restored in group* Solanum* + MeHg.

Histological evaluation of the male reproductive tract requires a good understanding of the form and function of the organs in order to distinguish changes in the normal morphology correlating with the animal reproductive status [22]. McNeil and Bhatnagar [37] showed that injury to seminiferous tubules and increased vacuolization were related to the dietary amount of MeHg. In accordance, our study showed that treated animals had degeneration of seminiferous tubules, absence of germ cells, and increased vacuolization in the seminiferous epithelium, possibly caused by a higher apoptotic index of germ cells caused by MeHg chronic exposure. At least part of these dead germ cells was seen in a higher incidence in the lumen of the epididymis of the MeHg-treated rats.

The possible increase in ROS production in the testis can cause significant damage to mitochondrial and nuclear DNA due to the alteration on the expression of genes such as Bcl-2 and Bax involved in molecular mechanisms promoters of germ cells apoptosis in response to oxidative stress [38]. Since MeHg increases the expression of these genes which promotes apoptosis, we observed a reduction in daily sperm production and this can be correlated with decreased testicular weight and histopathological changes observed. The reduced sperm number found in the epididymis reflected the lower DSP. On the other hand, sperm parameters in the* Solanum* plus MeHg group were comparable with the control group, indicating a possible protective effect of maná-cubiu. Facing the constant risk of oxidative stress which germ cells are subject to, which may impair reproductive physiology, the gonads need antioxidant protection during gamete production. Substances with antioxidant properties have achieved success in the treatment of infertility, such as maná-cubiu fruit, which exhibits antioxidant activity being able to decrease the production of reactive oxygen species.

This protective effect may be associated with possible antioxidant activity of niacin, a compound present in large quantities in fruit, which protects cell from DNA damage and can change the redox state induced by MeHg [5] or by the action of some chemical elements in the fruit, such as Zn, Fe, and Se. It is known that Zn and Fe participate of oxidation and reduction processes, and Zn as cofactors of Cu-Zn superoxide dismutase prevents deleterious effects of ROS on spermatozoa [39]. Thus, the improvements in histopathology, sperm production, and testicular weight may be related to coadministration of* Solanum* and MeHg.

The cytodifferentiation of the spermatozoa during spermiogenesis is responsible for morphological changes that define this specialized cell, and Zn deficiency may lead to degeneration of cells involved in spermatozoa processing after meiotic division [40]. Besides, at this stage, the germ cell loses large amount of their cytoplasm, and along with it, some of the enzymes of the antioxidant system, making these cells unable to regenerate from damage caused by ROS [41]. Therefore, abnormal sperm morphology has been associated with oxidative stress [42]. Fossato da Silva et al. [11] found a higher number of abnormal sperm in animals treated with MeHg, and this abnormality was mainly in the sperm head. Our results also showed a higher number of abnormalities in the sperm head caused by exposure to MeHg proving the deleterious action of reactive species in the normal development of spermatozoa. Once normal sperm morphology is better criterion for predicting fertility than sperm counts [43]; animals exposed to MeHg can present fertility problems.

The* Solanum* plus MeHg group showed an improvement in sperm morphology possibly due to maná-cubiu antioxidant activity. Once Zn directly influenced sperm morphology [44], it was demonstrated that low levels of Zn can increase percentages of broken flagellum [45]. In this study, we observed an improvement of flagellum morphology in* Solanum* group, showing that maná-cubiu utilization, rich in Zn, has a positive effect on sperm.

## 5. Conclusion

In conclusion, the present work confirmed previous results showing that chronic exposure to MeHg compromises GSH levels, sperm count, and quality, as well as the germinative epithelium of adult rats. Moreover, we also show the phytoremediation potential of maná-cubiu fruit, preventing deleterious effects caused by MeHg on the male reproductive system.

## Figures and Tables

**Table 1:** Body weight and reproductive organ weights of male rats.

Parameters	Control (*n* = 5)	MeHg (*n* = 5)	*Solanum * (*n* = 6)	*Solanum* + MeHg (*n* = 6)
Absolute weights				
Final body weight (g)	597.0 ± 5.7^a^	533.5 ± 13.9^b^	508.3 ± 10.0^b^	550.3 ± 18.3^a,b^
Testis (mg)	2,613 ± 87.02^a^	1,955 ± 15.23^b^	2,358 ± 86.92^a,b^	2,224 ± 33.83^a,b^
Epididymis (mg)	885.9 ± 30.2	755.5 ± 65.0	795.1 ± 35.8	779.3 ± 35.9
Ventral prostate (mg)	453.2 ± 35.4	434.7 ± 41.5	429.1 ± 55.0	542.3 ± 63.9
Full seminal vesicle (mg)	1,619 ± 182.3	1,592 ± 150.9	1,440 ± 66.7	1,500 ± 90.9
Empty seminal vesicle (mg)	630.9 ± 41.1	628.7 ± 71.7	586.5 ± 27.2	574.6 ± 32.8
Relative weights				
Testis (mg/100 g)	4.36 ± 0.14^a,b^	3.63 ± 0.20^a^	4.64 ± 1.74^b^	4.05 ± 2.42^a,b^
Epididymis (mg/100 g)	1.48 ± 0.04	1.40 ± 0.08	1.57 ± 0.09	1.42 ± 0.05
Ventral prostate (mg/100 g)	0.75 ± 0.05	0.80 ± 0.05	0.84 ± 0.10	0.98 ± 0.05
Full seminal vesicle (mg/100 g)	2.69 ± 0.28	2.94 ± 0.20	2.85 ± 0.18	2.72 ± 0.14
Empty seminal vesicle (mg/100 g)	1.05 ± 0.06	1.16 ± 0.10	1.16 ± 0.07	1.04 ± 0.05

Values expressed as mean ± SEM. One-way analysis of variance (ANOVA) test followed by Tukey test was performed. ^a,b^Mean values with the same letter do not differ statistically; *P* values < 0.05 were considered significant.

**Table 2:** Sperm morphology of control and treated animals.

Parameters	Control	MeHg	*Solanum *	*Solanum* + MeHg
Normal sperm	92.00 (91.50–93.38)^a^	75.00 (66.38–82.13)^b^	92.50 (86.63–94.38)^a^	89.75 (84.50–95.00)^a,b^
Abnormalities of the sperm head	6.00 (5.00–7.25)^a^	19.75 (12.75–31.13)^b^	6.50 (5.12–11.13)^a,b^	9.25 (2.37–13.00)^a,b^
Abnormalities of the flagellum	1.75 (0.37–2.12)^a,b^	4.00 (2.37–6.50)^a^	1.00 (0.50–1.75)^b^	2.00 (0.37–6.50)^a,b^
Presence of cytoplasmic droplet	15.00 (9.87–15.75)	19.00 (14.38–24.13)	23.00 (21.75–28.63)	34.00 (23.38–37.00)

*N* = 6/group. Values expressed as median and interquartile range (first and third). Kruskal-Wallis analysis of variance test followed by Dunn's test was performed. ^a,b^Medians with the same letter do not differ statistically; *P* values < 0.05 were considered significant.

**Table 3:** Sperm counts and transit time of male rats from control and treated groups.

Parameters	Control	MeHg	*Solanum *	*Solanum* + MeHg
Sperm head count (×10^6^/testis)	288.40 ± 8.72^a^	208.00 ± 24.11^b^	289.20 ± 12.08^a^	267.90 ± 18.83^a,b^
Sperm head count (×10^6^/g testis)	117.50 ± 4.59	112.00 ± 9.55	124.20 ± 9.22	121.4 ± 7.13
Daily sperm production (×10^6^/testis)	47.27 ± 1.43^a^	34.09 ± 3.95^b^	47.40 ± 1.98^a^	43.92 ± 3.09^a,b^
Daily sperm production (×10^6^/g testis)	19.26 ± 0.75	18.35 ± 1.56	20.35 ± 1.51	19.90 ± 1.17
Caput/corpus epididymis sperm count (×10^6^/organ)	236.70 ± 19.36^a^	155.30 ± 22.05^b^	195.80 ± 21.76^a,b^	175.90 ± 7.83^a,b^
Caput/corpus epididymis sperm count (×10^6^/g organ)	521.40 ± 26.61^a^	397.00 ± 34.22^b^	510.80 ± 26.78^a^	475.10 ± 12.61^a,b^
Transit time in the caput/corpus epididymis (days)	4.98 ± 0.29	4.49 ± 0.48	4.15 ± 0.43	4.12 ± 0.40
Cauda epididymis sperm count (10^6^/organ)	415.10 ± 25.26	285.50 ± 44.96	351.00 ± 36.33	339.80 ± 29.96
Cauda epididymis sperm count (10^6^/g organ)	1295.00 ± 50.68^a^	961.50 ± 59.96^a^	1116.00 ± 49.41^a,b^	1105.00 ± 20.14^a,b^
Transit time in the cauda epididymis (days)	8.77 ± 0.43	8.11 ± 0.41	7.34 ± 0.37	8.02 ± 1.12

*N* = 6/group. Values expressed as mean ± SEM. One-way analysis of variance (ANOVA) test followed by Tukey test was performed. ^a,b^Mean values with the same letter do not differ statistically; *P* values < 0.05 were considered significant.
